# Hemostatic and Wound Healing Properties of *Chromolaena odorata* Leaf Extract

**DOI:** 10.1155/2013/168269

**Published:** 2013-08-01

**Authors:** Hataichanok Pandith, Xiaobo Zhang, Jason Liggett, Kyung-Won Min, Wandee Gritsanapan, Seung Joon Baek

**Affiliations:** ^1^Department of Biomedical and Diagnostic Sciences, The University of Tennessee, Knoxville, TN 37996, USA; ^2^Department of Pharmacognosy, Faculty of Pharmacy, Mahidol University, 447 Sri Ayutthaya Road, Ratchathewi, Bangkok 10400, Thailand; ^3^Department of Biomedical and Diagnostic Sciences, College of Veterinary Medicine, University of Tennessee, 2407 River Drive, Knoxville, TN 37996-4542, USA

## Abstract

*Chromolaena odorata* (L.) King and Robinson (Siam weed) extract has been used to stop bleeding and in wound healing in many tropical countries. However, its detailed mechanisms have not been elucidated. In this study, we examined the molecular mechanisms by which Siam weed extract (SWE) affected hemostatic and wound healing activities. SWE promoted Balb/c 3T3 fibroblast cell migration and proliferation. Subsequently, we found that heme oxygenase-1 (HO-1), the accelerating wound healing enzyme, was increased at the transcriptional and translational levels by SWE treatments. The HO-1 promoter analyzed with luciferase assay was also increased by treatment of SWE in a dose-dependent manner. This induction may be mediated by several kinase pathways including MEK, p38MAPK, AKT, and JNK. Quantitative real-time PCR using undifferentiated promonocytic cell lines revealed that thromboxane synthase (TXS), a potent vasoconstrictor and platelet aggregator, was increased and MMP-9, an anti platelet aggregator, was decreased in the presence of SWE. Our studies presented that SWE accelerated hemostatic and wound healing activities by altering the expression of genes, including HO-1, TXS, and MMP-9.

## 1. Introduction

Wound healing is an intricate process in which usually the skin repairs itself after injury. The process is divided into four overlapping phases: hemostasis (cessation of bleeding), inflammation, proliferation, and remodeling [1]. Hemostasis is mainly controlled by thromboxane synthase (TXS), which converts prostaglandin H_2_ into thromboxane A_2_, a potent vasoconstrictor and platelet aggregator [2]. Plasminogen activator inhibitor type 1 (PAI-1) also plays a role in hemostasis by inhibition of fibrinolysis, which prevents failure of the hemostatic process [3]. Subsequently, neutrophils release free radicals to kill bacteria in the inflammation phase [4, 5], and heme and heme proteins also accumulate at the local site of the wound. These heme and heme proteins have prooxidative and proinflammatory properties by inducing the expression of adhesion molecules, causing vascular permeability and leukocyte infiltration. These actions initiate wound healing process. Heme oxygenase-1 (HO-1) has antiinflammatory and antioxidant activities and is responsible for a wide range of wound healing functions. It converts heme into biliverdin/bilirubin, iron and carbon monoxide, which are potent antioxidant products. The overexpression of HO-1 helps to accelerate wound healing such as amelioration of inflammation, proliferation and protection against endothelial cell apoptosis [6]. Matrix metalloproteinases (MMPs) also play a major role in wound healing by extracellular matrix (ECM) remodeling [7], and MMP-9 is key effector among those [8]. 

Siam weed (*Chromolaena odorata* (L.) King and Robinson) is a perennial scandent or semiwoody shrub in the Asteraceae family. It has been used for a variety of ailments in many tropical countries for a long time, especially to stop bleeding. Numerous studies have demonstrated that Siam weed extract (SWE) accelerates hemostasis [9–11] and wound healing [12–14]. However, the molecular targets of SWE in wound healing activity have not been identified. In this study, we investigated the effect of 70% ethanolic SWE (C1) and its bioactive compounds, scutellarin tetramethyl ether (scu) and stigmasterol, on enhanced wound healing activity. Scu and stigmasterol are the major components of SWE as assessed by thin-layer chromatography. In rat models, the 70% ethanolic extract possesses the highest effectiveness to stop bleeding, compared to other solvent extracts [15]. Scu and stigmasterol exhibit hemostatic [16] and antiinflammatory activities [17, 18]. 

In this study, we examined the molecular targets of SWE (C1) in the wound healing process and found that the expression of HO-1 and other proteins played a pivotal role in SWE-induced wound healing activity. Our data suggest that SWE affects the wound healing process by using novel molecular targets. 

## 2. Materials and Methods

### 2.1. Reagents

All antibodies were purchased from Santa Cruz Biotechnology (Santa Cruz, CA, USA). CellTiter 96 Aqueous One Solution Cell Proliferation Assay was purchased from Promega (Madison, WI, USA). Scutellarin tetramethyl ether (scu) and stigmasterol were purchased from ChromaDex Inc. (Irvine, CA, USA) and Sigma Aldrich (St. Louis, MO, USA), respectively. All chemicals were purchased from Fisher Scientific (Pittsburgh, PA, USA), unless otherwise specified. 

### 2.2. Preparation of Standard Solutions

12-O-tetradecanoyl-phorbol-13-acetate (TPA), scu and stigmasterol were dissolved in DMSO and were kept at −80°C until used. 

### 2.3. Plant Materials and Plant Extract Preparation

Siam weed mature leaves were collected from Samut Sakhon province, Thailand, in December 2009. The plant sample was identified by Dr. Wandee Gritsanapan, and the voucher specimen (no. CO-003) was deposited at the Department of Pharmacognosy, Faculty of Pharmacy, Mahidol University. The leaves were dried in a hot air oven at 60°C for 24 h. The dried sample was ground into moderate powder. Then, 100 g of the powder was exhaustively extracted with 70% v/v ethanol (EtOH) (1 : 10 w/v) while shaking at 25°C, 120 rpm for 12 h on a shaker. The mixture was filtered through Whatman filter paper no. 1, and the filtrate was concentrated using a rotary evaporator at 40°C. The dark green viscous ethanolic extract (C1) was kept in a tightly closed brown vial at 4°C until used.

A stock solution of the extract was prepared by complete dissolving of 342 mg of the ethanolic extract in 1 mL EtOH using sonication for 30 min. The stock solution was kept at −80°C until used. The working solutions were prepared by diluting the stock solution with EtOH to the concentrations of 3.42, 34.2 and 100 mg/mL. 

### 2.4. Cell Cultures

Balb/c 3T3 and U937 cells (ATCC, Manassas, VA, USA) were maintained in Dulbecco's modified Eagle's medium (DMEM) and RPMI 1640 medium, respectively. Both culture media were supplemented with 10% fetal bovine serum (FBS), penicillin G (100 U/mL), streptomycin (100 *μ*g/mL) and amphotericin B (0.25 *μ*g/mL) at 37°C and 5% CO_2_ in a humidified incubator.

### 2.5. Quantitative Real-Time-PCR and Reverse-Transcriptase PCR

U937 cells were grown to 80–90% confluence in 10 cm plates and treated with EtOH as vehicle, TPA 100 nM as positive control, SWE 34.2 and 100 *μ*g/mL, and scu 10 and 20 *μ*M, in the presence of 10% serum for 12 h. Total RNA was prepared using the Total RNA kit (Omega Bio-Tek, Norcross, GA, USA) and reverse transcripted with a verso cDNA kit (Thermo Scientific, Surrey, KT, USA) according to the manufacturers' instruction. Three replicates of real-time PCR were carried out using MyiQ thermal cycler (Bio-RAD, Hercules, CA, USA) for 25 cycles for human thromboxane synthase (*Tx*): sense strand 5′-GCCAAATGGAGCTCAGAAAG-3′, antisense strand 5′-TGCAGTAGCACCTCTGGATG-3′; human *PAI-1:* sense strand 5′-CACGAGTCTTTCAGACCAAGAG-3′, antisense strand 5′-CACACAAAAGCTCCTGTAAGC-3′; *MMP-9:* sense strand 5′-GCTCTTCCCTGGAGACCTG-3′, antisense strand 5′-ACACGCGAGTGAAGGTGAG-3′; and human *GAPDH:* sense strand 5′-GACCACAGTCCATGCCATCACT-3′, antisense strand 5′-TCCACCACCCTGTTGCTGTAG-3′ at 94°C for 30 s, 55°C for 30 s, and 72°C for 1 min using the ABsolute QPCR SYBR-Green mix (ABgene House, Epsom, UK) with specific primers for human *Tx*, *PAI-1, MMP-9, *and* GAPDH*. The quantification of each gene was calculated. For the reverse-transcriptase PCR, the reaction was carried out for 25 cycles for mouse *HO-1:* sense strand 5′-CCCACGCATATACCCGCTAC-3′, antisense strand 5′-CTAGCAGGCCTCTGACGAAG-3′ and mouse *GAPDH: *sense strand 5′-CAGGAGCGAGACCCCACTAACAT-3′, antisense strand 5′-GTCAGATCCACGACGGACACATT-3′ at 94°C for 30 s, 55°C for 30 s, and 72°C for 1 min using ReadyMix Tag polymerase (Sigma Aldrich) with specific primers. Each PCR product was electrophoresed on 1% agarose gel and visualized by ethidium bromide staining.

### 2.6. Cell Migration Assay

Balb/c 3T3 cells were grown in DMEM supplemented with 10% FBS until confluence in 6 cm plates. The cells were starved with DMEM in an absence of serum for 24 h. Cell monolayer was scratched with a sterile pipette tip. Any cellular debris was removed by washing with 1 X phosphate buffer saline (PBS), and media were replaced with 1% DMEM containing EtOH as a control, and 3.42, 34.2 and 100 *μ*g/mL of SWE. The cells were viewed using an Olympus IMT2 microscope (Lake Success, NY, USA) at 40x total magnification and captured using a Sony model XC-ST50 camera (Park Ridge, NJ, USA) at 0, 6, 12 and 24 h. The degree of cell migration was observed by the gap between control and treatment. 

### 2.7. Cell Proliferation Assay

The effect of SWE on cell proliferation in Balb/c 3T3 cells was investigated using the CellTiter 96 Aqueous One Solution Cell Proliferation Assay (Promega). Cells were seeded at a density of 2.0 × 10^4^ cells/well in 96-well tissue culture plates in four replicates and grown for 24 h. Cells were then treated with EtOH, 3.42, 34.2 and 100 *μ*g/mL of SWE in the presence of 10% serum. At 12 h after treatment, 20 *μ*L CellTiter 96 Aqueous One Solution was added to each well, and the plate was incubated for 1 h at 37°C. Absorbance at 490 nm was recorded in an enzyme-linked immunosorbent assay (ELISA) plate reader (Bio-Tek Instruments, Winooski, VT, USA). 

### 2.8. Western Blot Analysis

Balb/c 3T3 cells were grown to 80–90% confluence in 6 cm plates. The cells were starved for 24 h before treatment with 3.42, 34.2 and 100 *μ*g/mL SWE; and Siam weed's bioactive compounds scu and stigmasterol 10 and 20 *μ*M, using EtOH or DMSO as vehicle controls in the absence of serum for 12 h. Total cell lysates were isolated using RIPA buffer (1 x PBS, 1% NP-40, 0.5% sodium deoxycholate, and 0.1% SDS) supplemented with protease inhibitors (1 mM phenylmethylsulfonyl fluoride, 5 *μ*g/mL aprotinin and leupeptin) and phosphatase inhibitors (1 mM Na_3_VO_4_ and 1 mM NaF). Protein concentration was determined by the BCA protein assay (Pierce, Rockford, IL, USA). Protein (30 *μ*g) was separated on sodium dodecyl sulfate-polyacrylamide gel and transferred into a nitrocellulose membrane (Pall Life Sciences, Pensacola, FL, USA). The blots were blocked with 5% skim milk/Tris buffer saline/Tween 0.05% (TBS-T) for 1 h and probed with a specific primary antibody in 5% skim milk (HO-1 antibody was hybridized with 1% skim milk)/0.05% TBS-T at 4°C overnight. After three washes with TBS-T, the blots were incubated with horseradish peroxidase-conjugated secondary antibody for 1 h and washed with TBS-T several times. Proteins were detected by the enhanced chemiluminescence system (Thermo Scientific, Rockford, IL, USA). 

### 2.9. Transient Transfection and Luciferase Promoter Assay

Transient transfection was performed using *Trans*IT-LT1 (Mirus Bio LLC., Madison, WI, USA) according to the manufacturer's protocol. Briefly, Balb/c 3T3 cells were plated in 12-well plates at a density of 2.0 × 10^5^ cells/well and grown to 50% confluence. The plasmid *pHO-1GL3/9.4* luciferase construct was kindly provided by Dr. Anupam Agarwal (University of Alabama at Birmingham). The plasmid mixtures containing 1 *μ*g/mL *HO-1*/*9.4-Luc* plasmid and 0.1 *μ*g/mL *pRL-null* vector were cotransfected for 24 h. Transfected cells were pretreated with 3.42, 34.2 and 100 *μ*g/mL of SWE, 10 and 20 *μ*M of scu compound, and 10 *μ*M of stigmasterol for 24 h in the presence of 10% serum. EtOH and DMSO were used as vehicle controls for extract and bioactive compounds, respectively. Cells were harvested in 1 x luciferase lysis buffer (Promega, Madison, WI, USA), and luciferase activity was measured and normalized to the *pRL-null* luciferase activity using a dual luciferase assay kit (Promega, Madison, WI, USA).

### 2.10. Statistic Analysis

Statistical significance was calculated using the Newman-Keuls Multiple Comparison Test comparing all pairs of means (*P* < 0.05).

## 3. Results and Discussions 

### 3.1. SWE Promotes Fibroblasts Cell Migration and Proliferation

**Figure 1:**
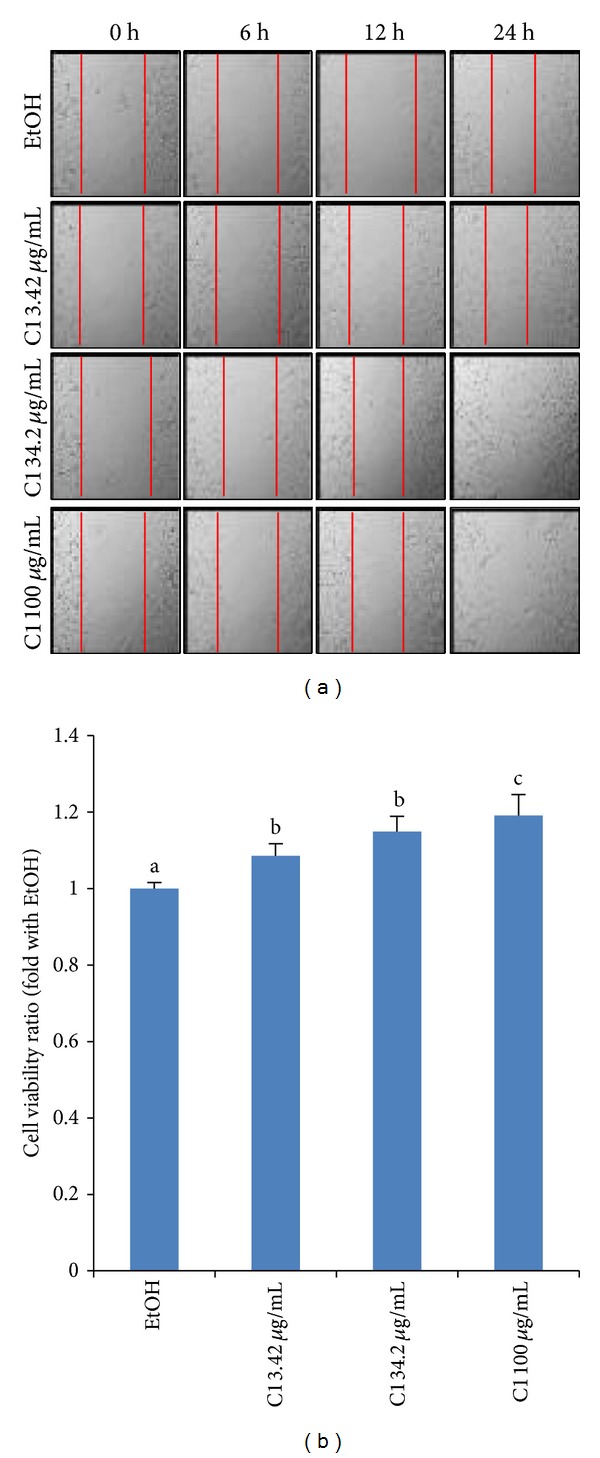
Cell migration and proliferation by SWE. (a) Balb/c 3T3 fibroblasts were scratched using a pipette tip to make gaps between cells before treatment. At 6, 12, and 24 h of treatment, the plates were photographed under a light microscope (40x). This picture is representative of five independent fields. It is notable that 100 *μ*g/mL SWE for 24 h is toxic to the cells. (b) Balb/c 3T3 cell proliferation was measured using the CellTiter96 Aqueous One Solution Cell Proliferation Assay. Values are expressed as mean ± SD of four replicates. Different letters (a, b, and c) represent statistically significant differences of means between groups (*P* < 0.05).

In wound healing, cell migration is an important factor, that is, directed and modulated by a wide range of cellular signals. Among them, chemical stimuli have been shown to play significant roles in guiding the direction of locomotion and in the accumulation of cells. To investigate the wound healing activity of SWE, we employed fibroblast cell lines (Balb/c 3T3 cells) to perform cell migration and proliferation assays. As shown in Figure 1(a), SWE facilitated cell migration of Balb/c 3T3 fibroblasts, compared to EtOH-treated cells. The gap between cells was nearly closed after 24 h with 34.2 *μ*g/mL of SWE, compared to gap in vehicle-treated cells which was still significant after 24 h. However, the cells began to die off when 100 *μ*g/mL SWE was added for 24 h. Proliferation of Balb/c 3T3 fibroblasts was analyzed to evaluate cellular responses to SWE. SWE modestly but significantly stimulated cell proliferation with a strong response at the doses of 34.2 and 100 *μ*g/mL (Figure 1(b)). Interestingly, 100 *μ*g/mL SWE with 1% serum caused cell death in a scratch assay, whereas 100 *μ*g/mL SWE with 10% serum facilitated cell growth. Cell migration and proliferation are important mechanisms of tissue regeneration by which SWE facilitates the wound healing process.

### 3.2. SWE Increased HO-1 Protein and mRNA

**Figure 2:**
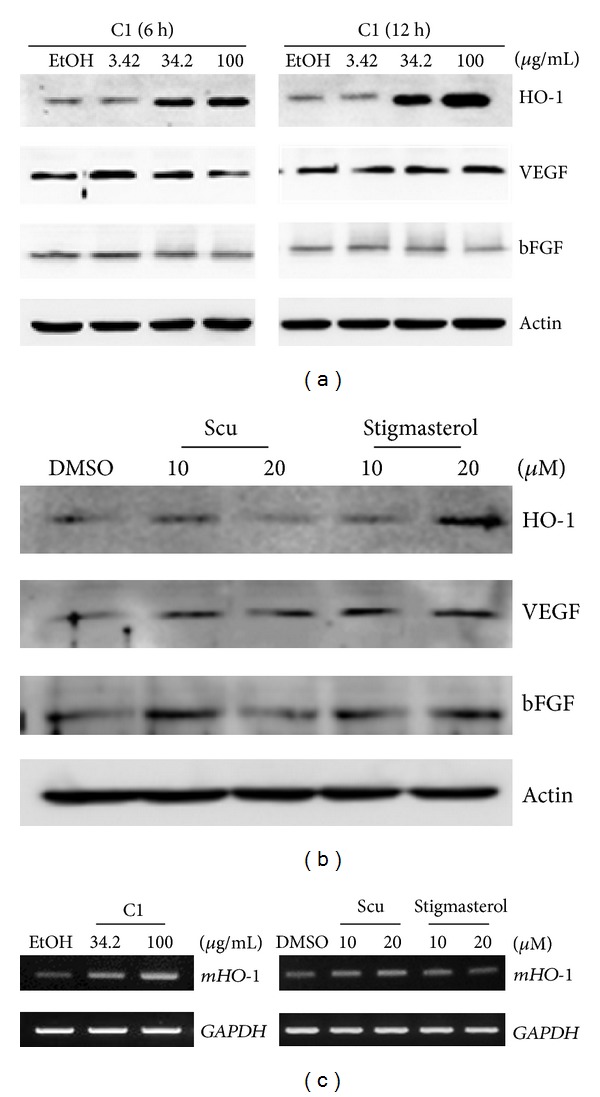
Expression of genes involved in wound healing activity. (a) Balb/c 3T3 fibroblasts were grown in the presence of indicated SWE for 6 h and 12 h. The cell lysates were electrophoresed in SDS-PAGE, and several genes and proteins were examined. Actin was used as a control. (b) Scu and stigmasterol were added to Balb/c 3T3 cells for 12 h. Western blot was carried out as described in the Materials and Methods section. (c) Balb/c 3T3 cells were treated with SWE for 12 h and HO-1 mRNA expression was measured. Reverse-transcriptase PCR was performed, and the data represent three independent experiments. GAPDH was used as control.

To determine molecular targets of SWE in the wound healing process, transcription factors and angiogenesis factors were examined because they are involved in the wound healing process. VEGF and bFGF stimulates wound healing through angiogenesis. The VEGF also promotes collagen deposition and epithelialization [19]. Treatment with SWE and its major bioactive components was done to examine the expression of these proteins. As shown in Figure 2(a), SWE at 34.2 and 100 *μ*g/mL increased HO-1 expression, but VEGF and bFGF expressions were not changed. We observed that HO-1 expression increased in a dose-dependent manner, with the highest expression at the 100 *μ*g/mL dose for 12 h treatment. We also measured two bioactive components in SWE, scu and stigmasterol, and found that only HO-1 protein expression increased with 20 *μ*M of stigmasterol (Figure 2(b)). Further HO-1 mRNA expression was also investigated. As a result, we found that SWE increased HO-1 mRNA in a dose-dependent manner, whereas scu and stigmasterol did not affect mRNA expression (Figure 2(c)). It has been shown that HO-1 is necessary for efficient wound closure and neovascularization [20]. Thus, our results suggest that SWE facilitated the wound healing process through the induction of HO-1 expression. The antioxidative activity of HO-1 is one of pathway which accelerates wound healing. The antioxidative effect of SWE against oxidizing agents on human dermal fibroblasts and epidermal keratinocytes [21] might come from the increasing of HO-1 level. Interestingly, SWE increased HO-1 at the transcriptional level, whereas stigmasterol may affect HO-1 expression at the translational level. It has been reported that SWE and its flavonoids exhibited inhibitory activity of platelet-activating factor (PAF) receptor binding [22]. The PAF is important mediator in the inflammatory response. Therefore, SWE and its flavonoids could play a role in hemostatic and wound healing activity via PAF pathway, but further analysis remains to be investigated. 

### 3.3. SWE Increased HO-1 Promoter Activity

**Figure 3:**
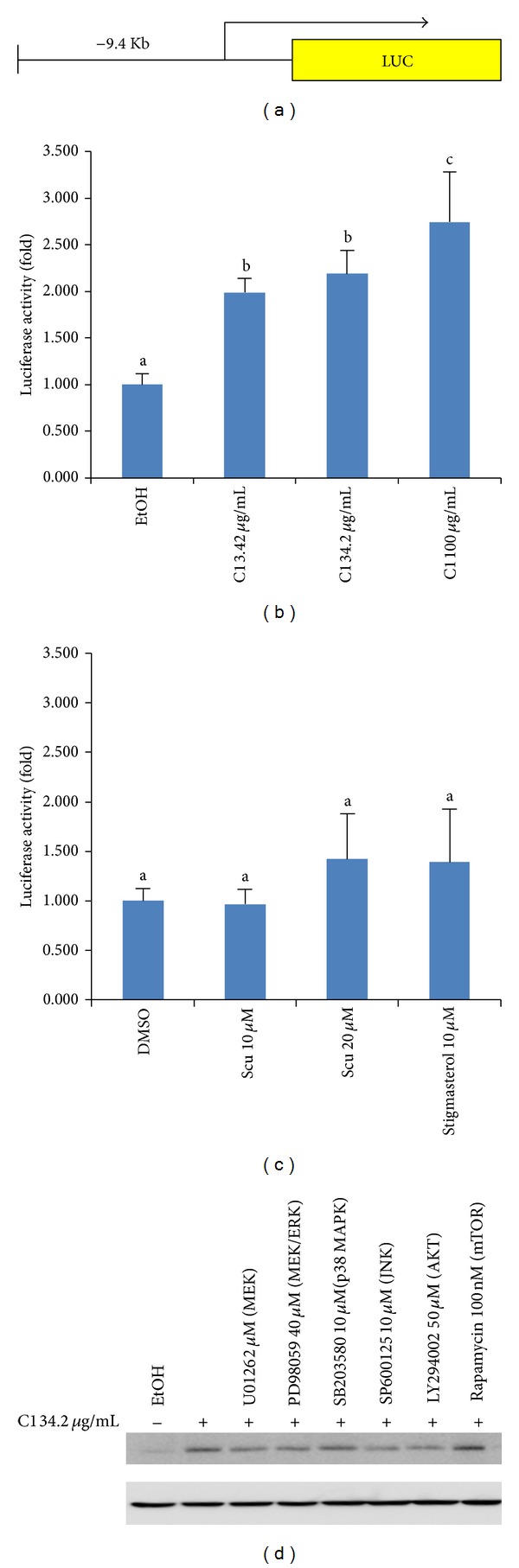
SWE-induced HO-1 expression. (a) Schematic diagram of human HO-1 promoter. HO-1 promoter construct was transfected into fibroblast cells, and luciferase was measured after treatment of indicated SWE (b) or single compound (c). Values are expressed as mean ± SD of four replicates. Different letters (a, b, and c) represent statistically significant difference of means compared to the values of controls and compared between groups (*P* < 0.05). (d) Western blot analysis of HO-1 expression in the presence of kinase inhibitors. Actin was used for control. The cells were pretreated with indicated kinase inhibitors for 1 h, followed by SWE treatment for 12 h. Cell lysates were subjected to Western blot.

To further provide evidence that SWE affects HO-1 expression at the transcriptional level, we examined the promoter activity of HO-1. As shown in Figure 3(a), 9.4 kb HO-1 promoter was used to test HO-1 activity in fibroblasts. All concentrations of SWE increased HO-1 promoter activity; however, single compounds did not affect promoter activity (Figures 3(b) and 3(c)). These results are consistent with the mRNA expression shown in Figure 2(c). To further elucidate signaling pathways affected by SWE-induced HO-1 expression, we treated cells with different kinase inhibitors in the presence of SWE. The result revealed that many kinase pathways could affect SWE-induced HO-1 expression to varying degrees except for the mTOR pathway (Figure 3(d)). Indeed, our result is consistent with a recent report indicating that HO-1 is regulated by AKT, p38MAPK, and JNK [23].

### 3.4. SWE Affected the TXS, PAI-1, and MMP-9 mRNA Production

**Figure 4:**
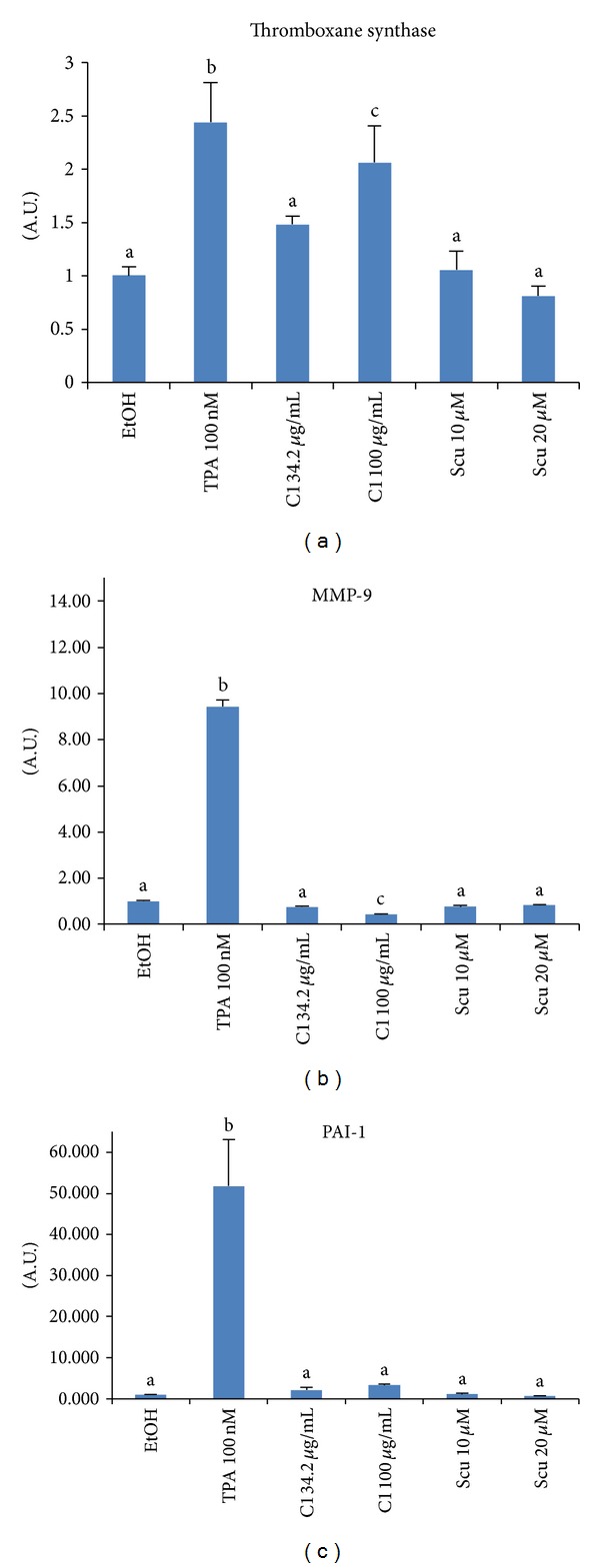
Expression of (a) TXS, (b) MMP-9, and (c) PAI-1 in U937 cells. U937 cells were treated with SWE, TPA, or scu for 12 h, and gene expression was analyzed by real-time PCR performed using a specific primer described in Section 2. Values are expressed as mean ± SD of four replicates. Different letters (a, b, and c) represent statistically significant difference of means compared to the values of controls and compared between groups (*P* < 0.05).

Human promonocytic cell line U937 is well known to be differentiated by 12-*O*-tetradecanoyl-phorbol-13-acetate (TPA or PMA), accompanied with increased expression of TXS [24], PAI-1 [25], and MMP-9 [26]. Since these genes play an important role in wound healing, we measured alterations of them in the presence of SWE. U937 cells were treated with either TPA as a control or compounds, and TXS, PAI-1, and MMP-9 genes were examined by real-time PCR. As shown in Figure 4(a), SWE at the dose of 100 *μ*g/mL stimulated TXS mRNA expression without TPA, but PAI-1 expression was not altered by compounds (Figure 4(b)). Interestingly, MMP-9 mRNA expression was decreased in the presence of 100 *μ*g/mL SWE (Figure 4(c)). It has been shown that MMP-9 is not only a wound healing stimulator, but also an antiplatelet aggregator by inhibition of thrombin and collagen-induced platelet aggregation [27, 28]. Moreover, increased MMP-9 expression predicts poor wound healing [29]. Thus, MMP-9 may be involved in wound healing activity by SWE as an antiplatelet aggregator. Neither scu nor stigmasterol, altered the mRNA expression of these three genes.

## 4. Conclusions

**Figure 5:**
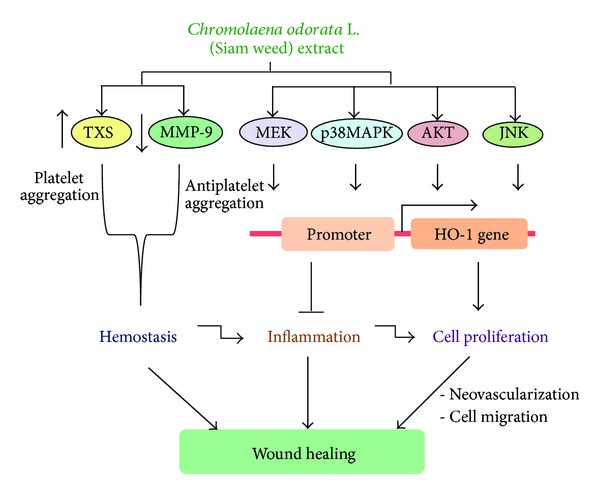
Schematic diagram showing that *C. odorata* extract stimulated the hemostatic activity by stimulation of TXS and repression of MMP-9 expressions. It also activates MEK, p38MAPK, AKT and JNK kinase pathways which initiated the expression of HO-1. The induction of HO-1 will inhibit an inflammation and stimulate cell proliferation, thereby enhancing neovascularization and cell migration that helps to accomplish wound healing.

In summary, our results indicate that SWE facilitated wound healing activity as assessed by cell migration and proliferation assays. This wound healing activity may be mediated by HO-1 induction in fibroblasts, whereas TXS induction and MMP-9 suppression in U937 cells may also contribute to SWE-induced wound healing activity (Figure 5).
